# The Robotic Lumbar Spine: Dynamics and Feedback Linearization Control

**DOI:** 10.1155/2013/985248

**Published:** 2013-09-16

**Authors:** Ernur Karadogan, Robert L. Williams

**Affiliations:** ^1^Mechanical Engineering Department, University of Texas-Pan American, Edinburg, TX 78539, USA; ^2^Mechanical Engineering Department, Ohio University, Athens, OH 45701, USA

## Abstract

The robotic lumbar spine (RLS) is a 15 degree-of-freedom, fully cable-actuated robotic lumbar spine which can mimic *in vivo* human lumbar spine movements to provide better hands-on training for medical students. The design incorporates five active lumbar vertebrae and the sacrum, with dimensions of an average adult human spine. It is actuated by 20 cables connected to electric motors. Every vertebra is connected to the neighboring vertebrae by spherical joints. Medical schools can benefit from a tool, system, or method that will help instructors train students and assess their tactile proficiency throughout their education. The robotic lumbar spine has the potential to satisfy these needs in palpatory diagnosis. Medical students will be given the opportunity to examine their own patient that can be programmed with many dysfunctions related to the lumbar spine before they start their professional lives as doctors. The robotic lumbar spine can be used to teach and test medical students in their capacity to be able to recognize normal and abnormal movement patterns of the human lumbar spine under flexion-extension, lateral bending, and axial torsion. This paper presents the dynamics and nonlinear control of the RLS. A new approach to solve for positive and nonzero cable tensions that are also continuous in time is introduced.

## 1. Introduction

Teaching art of palpation to medical students is a challenging task. In institutions that teach palpatory diagnosis, it is taught by using voluntary human patients who are mostly palpated by the instructor for demonstrative purposes. Meanwhile, the students usually watch the process and get to palpate only their lab partners as “patients” who are, considering the general population of medical students, relatively young and healthy (many with limited dysfunctions). It is, however, very difficult to be able to find and demonstrate a different patient for every single dysfunction that the students are taught during the lectures or in the laboratories. Therefore, it is still hard to teach and learn palpatory diagnosis for different variations of dysfunctions. The lack of a means for evaluating the transfer of practical information from the instructor to the students is another drawback that the medical schools are facing today. There exists no assessment device for instructors to objectively evaluate progress and success of the students in real-life situations.

The need for a “gold standard” to objectively assess the palpation accuracy is apparent. The design of such a device has the potential of becoming a standardized means for training medical students since the repeatability of many dysfunctions would be possible. Repeatability is a main concern in real-life medical education situations, because the properties of human soft tissue (stiffness, tenderness, etc.) can alter when it is touched by the examiner. The tissue properties are not the same even between the beginning and end of an examination. A legitimate method of evaluating the students would be comparing the first diagnosis of the instructor with the diagnosis of the student. However, when the student takes over the patient, he/she tries to diagnose movement patterns and/or the tissue properties that have already been changed due to the stimulation of the instructor.

The role of simulation in medical education is rapidly increasing. The simulations to train nurses, veterinarians, and doctors (osteopathic and allopathic) have been and are still being developed due to their effectiveness and cost-reducing advantages. These simulations can be computer based or in the form of mannequins that can simulate some functions of the real human body such as breathing and blood pressure. Computer-based haptic simulations require the utilization of a haptic interface to interact with the virtual objects inside a computer screen. That is clearly not the case when humans really interact with real objects. For instance, the VHB [1], the only simulation that is being used to improve palpatory skills of medical students, simulates somatic dysfunctions by increased stiffness of an area on the virtual back and the users “touch” the back with PHANToM haptic devices which only stimulate the proprioceptive receptors and introduces an extra layer of disturbance between the fingers and the computer-generated objects to be sensed. Therefore, a simulation system which allows the user to interact with a real object would be a better and more effective approach.

The robotic spine concept has been studied over the past years [2–4]. These studies built humanoid robots with a flexible spine which would enhance the human-like movements of the robots and increase the range of movement of the robot's torso. These humanoid robots dealt with the movement of the whole spine, rather than the relative position and stiffness of a vertebra with respect to the adjacent ones. They sufficiently accomplished flexible spine movements with less than the total number of vertebrae in a human spine. However, no research has yet been completed on the subject of developing a robotic spine with anatomically accurate vertebrae geometry and movements for tactile medical education and/or proficiency assessment. In this paper, the dynamic model of a robotic lumbar spine is derived and used in designing a nonlinear controller. A new method to solve for positive and nonzero cable tensions that are also continuous in time is introduced. Simulations to test the controller for the RLS are presented. In the RLS, individual vertebra is controlled by four cables that are attached to four motors. In this case, a cable-actuated robot is practical due to the space limitations between vertebrae. The robot will be controlled by a joystick or autonomously by preprogramming. The user will interact by touching the posterior aspect of the lumbar spine that is covered with a skin-like material. The user will try to find the type and region of the dysfunction by comparing the movement patterns at different configurations of the robotic lumbar spine. In this current form, the RLS will be capable of training users in terms of healthy and dysfunctional movements of the lumbar spine. Addition of the capability to adjust (rotational) stiffness of the vertebrae associated with normal and abnormal rotational limits is also underway. This will enable users to train on anatomically normal (abnormal) movement patterns as well as feel normal (abnormal) joint stiffness associated with a healthy (dysfunctional) lumbar spine.

## 2. Construction of the Lumbar Spine Geometry

**Figure 1:**
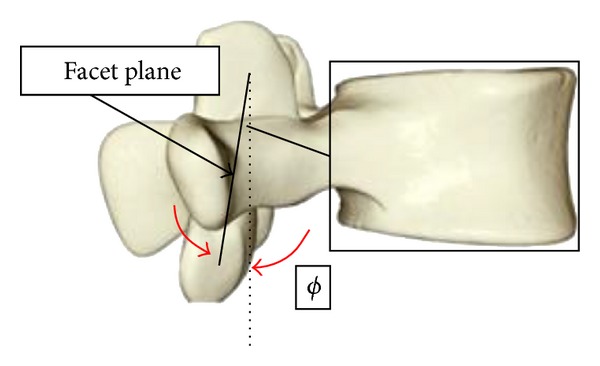
Facet plane and angle.

**Figure 2:**
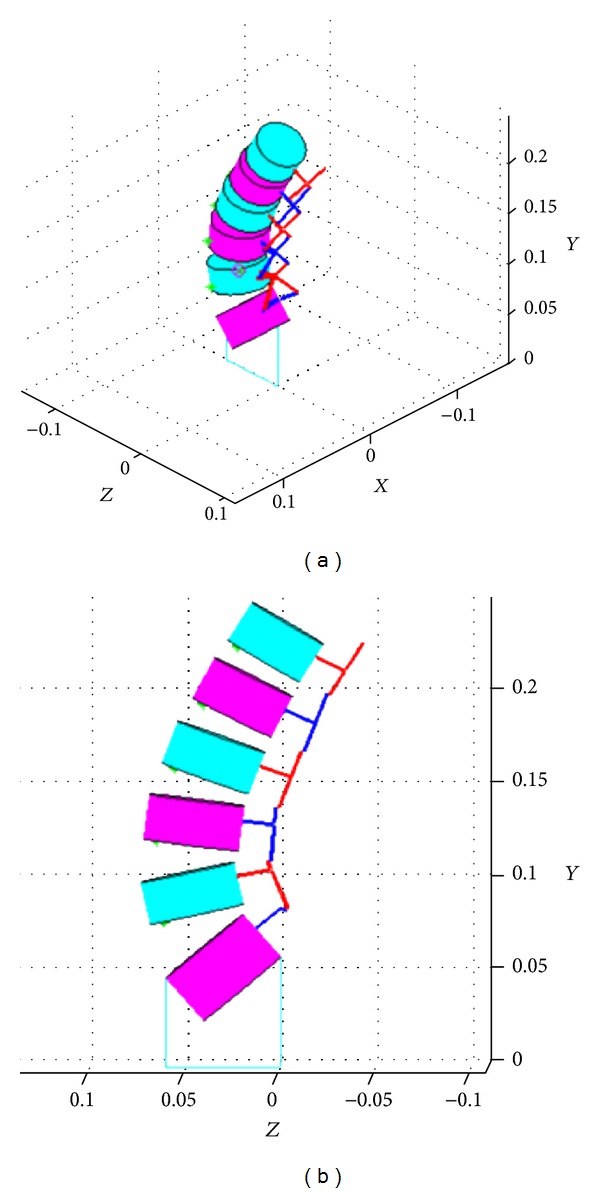
Three-dimensional geometry of the lumbar spine [6].

The geometry of the lumbar spine was constructed using dimensions of an average human spine based on published experimental data [5]. All parameters used for the reconstruction of the geometry except for the facet plane and facet plane angle (*φ*) have been previously used in the literature and measured to define the morphology of the vertebrae. We, assuming sagittal symmetry, define a facet plane as the plane that connects the centers of the four facets (left/right, superior/inferior) of a vertebra. This plane (manufactured as a plate) will allow the attachment of posterior elements with various dimensions on the same vertebral body making the system modular. The facet plane angle is defined to be the angle between the facet plane and the posterior wall of the vertebral body. In modeling, a cylindrical shape is assumed for the vertebral bodies.  Figure 1 shows the facet plane angle and the approximation of the vertebral bodies as cylinders. The constructed lumbar spine geometry is shown Figure 2. A detailed explanation on the construction of the lumbar spine geometry can be found in [6].

## 3. 3D Static Model of the Human Lumbar Spine

In order to design a device that mimics an average adult's lumbar spine, it is necessary to have anatomically correct movement patterns of each lumbar vertebra. In this study, these movement patterns were acquired by using a three-dimensional static model of the human spine. The mathematical model includes five lumbar vertebrae and the sacrum, elastic elements that connect inferior facets of one vertebra to the superior facets of the lower one and torsion springs that represent the collective torque resisting effects of the intervertebral disc and ligaments. It has been shown with several studies [5, 7–9] that the significant motion of the vertebrae during the movement of the spine is the rotational motion. Therefore, a spherical joint is chosen to connect each vertebra to the lower one. The location of this joint is critical in order to provide anatomically correct motion for each vertebra during the movement of the entire lumbar spine. In this model, the spherical joints are located at the inferoposterior corners of the vertebral bodies since the experimental data used for validation is based on the findings from [5]. The complete details including derivation and validation of this model can be found in [10].

## 4. RLS Kinematics

**Figure 3:**
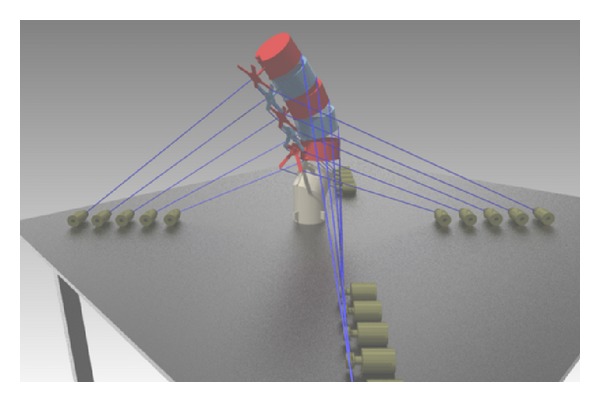
The robotic lumbar spine (RLS).

The robotic spine, shown in Figure 3, was designed based on the study by [5] since it details how lumbar spine moves in prespecified loading conditions. In that study, the upper-most vertebrae of freshly frozen cadaveric human lumbar spines with no abnormalities were exposed to external pure moments in order to induce motion, and both rotational and translational movements of each vertebra were recorded. 

The RLS is actuated by 20 cables connected to electric motors. Every vertebra is connected to the neighboring vertebrae by spherical joints. The use of spherical joints is intentional since the rotational motion of the vertebrae is more prominent as compared to their translational motion. The location of the spherical joint for each vertebra is at the inferoposterior corner in the mid-sagittal plane of the vertebral body. These locations correspond to the origin of the coordinate frames with respect to which the angles of rotation were recorded in [5]. As discussed previously, the facet plane in Figure 1 was designed to be used as the base on which posterior elements with various dimensions can be attached.

The cable connection points on the ground are at the corners of five trapezoids. The innermost trapezoid that includes the connection points for the fifth lumbar vertebra (L5) has posterior base length of 0.4 m, anterior base length of 0.2 m, and height of 0.15 m. The remaining four trapezoids are constructed with increasing the height of the adjacent (inner) one by 0.05 m anteriorly and 0.05 m posteriorly. This placement of the cable connections on the ground prevented cable interference during the simulations for six motion types: flexion/extension, right/left bending, and right/left axial torque [6].

## 5. RLS Dynamics and Control

### 5.1. Dynamic Model

In this section, we will develop the dynamic equations for the robotic lumbar spine. The dynamic equations can be stated in the following general format:

(1) $$\begin{array}{c} M\left(q\right)\ddot{q}+V\left(q,\dot{q}\right)+G\left(q\right)=\tau , \end{array}$$

where *q* is the vector of generalized coordinates (joint variables), $\dot{q}$ is the vector of generalized velocities, **M** is the inertia matrix as a function of *q*, **V** is the Coriolis/centripetal term as a function of *q* and $\dot{q}$, **G** is the gravity term as a function of *q* and **τ** is the vector of generalized forces that are found by using the forces applied by the cables. Note that, for systems the links of which are actuated at the joints, **τ** is independent of *q* when they are defined to be the joint variables (angle of rotation for revolute and distance for prismatic joints). However, as shown later in the text, **τ** for the robotic lumbar spine are functions of the generalized coordinates as well. The friction is neglected during the dynamic modeling stage.

We will use the energy-based Lagrange equation to derive the equations of motion for the robotic spine. The Lagrange formulation does not require the knowledge of the constraint forces when all of the constraints in a system are *holonomic* [11]. In Newton-Euler formulation, however, the constraint forces between adjacent links must be included as variables. The robotic lumbar spine is composed of only rigid bodies that are connected to each other via spherical joints. That is, all constraints in the system are *holonomic* constraints. The Lagrange equation can be stated as

(2) $$\begin{array}{c} \frac{d}{dt}\left(\frac{dL}{d\dot{q}}\right)-\frac{dL}{dq}=\tau , \end{array}$$

where *L* is the Lagrangian and defined as the difference between the kinetic and potential energy

(3) $$\begin{array}{c} L\left(q,\dot{q}\right)=K\left(q,\dot{q}\right)-U\left(q\right). \end{array}$$

Since the potential energy is not a function of the generalized velocities, the Lagrange equation for the *i*th generalized coordinate can be written as

(4) $$\begin{array}{c} \frac{d}{dt}\left(\frac{\partial K}{\partial {\dot{q}}_{i}}\right)-\frac{\partial K}{\partial q_{i}}+\frac{\partial U}{\partial q_{i}}=\tau_{i}. \end{array}$$

By using the chain rule, the above equation can also be written as

(5) ∑j=1n(d(∂K/∂q˙i)dqjq˙j+d(∂K/∂q˙i)dq˙jq¨j)−∂K∂qi+∂U∂qi=τi,

where *n* is the total number of generalized coordinates.

Adapting to the general format of the dynamic equations (1), **M**(*q*), $V\left(q,\dot{q}\right)$ and **G**(*q*) can be deducted from (5) as:

(6) M(q)=[m(q)11⋯m(q)1j⋮⋱⋮m(q)i1⋯m(q)ij],V(q,q˙)=[v(q,q˙)1⋮v(q,q˙)i],G(q)=[g(q)1⋮g(q)i],

where

(7) m(q)ij=d(∂K/∂q˙i)dq˙j,v(q,q˙)i=∑j=1n(d(∂K/∂q˙i)dqjq˙j)−∂K∂qi,gj(q)=∂U∂qj=∂(−migTTiBPCGii)∂qj=∑i=1Nv−migT∂(TiB)∂qjPCGii,

where *m*_*i*_ is the mass, ^*B*^ **P**_*CG*_*i*__ is the augmented vector involving center of gravity coordinates of the *i*th vertebra, $g={\left[\begin{array}{cccc} g_{x} & g_{y} & g_{z} & 0 \end{array}\right]}^{T}={\left[\begin{array}{cccc} 0 & -9.806 & 0 & 0 \end{array}\right]}^{T}$ is the augmented (a zero is added as the last element) gravitational acceleration since *y*-axis is directed upward and [_*i*_^*B*^ *T*] = [_1_^*B*^ *T*][_2_^1^ *T*] ⋯ [_*i*_^*i* − 1^ *T*] is the 4 × 4 homogenous transformation matrix that represents *i*th vertebra coordinate system with respect to the base frame {*B*}. The transformation matrix of a frame with respect to the neighboring one is expressed as:

(8) [Ti+1i]4×4=[[Ri+1i]3×3{Pi(i+1)ORG}3×10    0    01].

Total kinetic energy of the RLS is:

(9) K=∑1NvKii,

where *N*_*v*_ is the total number of vertebrae and ^*i*^ *K*_*i*_ is the kinetic energy of the *i*th vertebra expressed in the local vertebral frame and defined as

(10) Kii=12mViGi·ViGi+12 iωi·HiGi,

where ^*i*^ **V**_*G*_*i*__ is the linear velocity of the center of gravity, ^*i*^ **ω**_*i*_ is the angular velocity, ^*i*^ **H**_*G*_*i*__ = ^*i*^ **I**_*G*_*i*__^*i*^ **ω**_*i*_ is the angular momentum of the *i*th vertebra with respect to its local frame, and ^*i*^ **I**_*G*_*i*__ is the inertia tensor.

The right hand side of (2) is composed of the generalized cable forces and the partial derivative of the potential energy with respect to joint variables (*q*) which is actually the gravity term, **G**(*q*), in the Lagrange equation. Note that **τ** is the vector of generalized cable forces. The resulting generalized forces must be calculated for proper use of the Lagrange equation. The *k*th generalized force for the robotic lumbar spine can be written as

(11) τk=∑i=1Nv∑j=1NctijT∂(TiBPiji)∂qk=∑i=1Nv∑j=1NctijT∂(TiB)∂qkPiji=∑i=1Nv∑j=1NctijL^ijT∂(TiB)∂qkPiji=A(q)15×20{t}20×1,

where ^*i*^ **P**_*ij*_ is the augmented position vector from the origin of the local coordinate frame of the *i*th vertebra to the connection point of the *j*th cable in {*i*}, ${\hat{L}}_{ij}$ is the unit vector in the corresponding cable direction, and [_*i*_^*B*^ *T*] is the previously defined 4 × 4 homogenous transformation matrix.

### 5.2. Cable Tension Optimization

One of the challenges of designing a cable-actuated robot is the fact that the cables must be in tension (positive) at all times during the operation of a task. The robots with rigid links that are actuated with motors are not subject to this limitation. The RLS, being a fully cable-actuated robot, needs to be supplied with positive cable tensions. We start with the previously derived dynamics equations (independent variables are not shown for clarity):

(12) M(m×m)q¨(m×1)+V(m×1)+G(m×1)=A(m×n)t(n×1),

where *m* is the number of degrees of freedom (=15) and *n* is the number of cables (=20). In order to solve for positive cable tensions we introduce an intermediate variable **τ**_*v*_, which is the virtual input [12] to the system and defined as

(13) τv(n×1)=Mq¨+V+G.

Therefore, the dynamics equation can be written as:

(14) $$\begin{array}{c} \tau_{v}=At. \end{array}$$

Equation (14) can be solved by:

(15) t=A+τv+(Im−A+A)z,

where **A** ^+^ = **A** ^*T*^(**A** **A** ^*T*^)^−1^ is the Moore-Penrose pseudo-inverse of **A**, **I**_*m*_ is the (*m* × *m*) identity matrix, the first term on the right hand side is the particular solution and the second term is the homogenous solution which maps **z** (*m* × 1 vector) to the null space of **A**. The homogenous solution can take any value making the solution nonunique. This property can be utilized to search for a solution that will generate positive cable tensions that are needed to control the RLS. On the other hand, when the homogenous solution is zero, the tensions are calculated in the least-squares sense which does not guarantee a solution that will satisfy the positive cable tensions criterion. It is also imperative to obtain positive and nonzero cable tensions in order to be able to keep the robot under control during a task. Equation (15) can also be written as [13]:

(16) t=A+τv+N(A)σ,

where *N*(**A**)  is the (*n* × *n* − *m*) kernel matrix of **A** and **σ**_(*n*−*m*×1)_ = {*σ*_1_, *σ*_2_,…,*σ*_*n*−*m*_}^*T*^ is an arbitrary vector.

An optimization procedure can be employed with a proper objective function to solve (16) with positive and nonzero cable tensions. One of the optimization procedures is by using linear programming, which is formulated as [14]

(17) min⁡σ fTσ,such  that t=A+τv+N(A)σ,        −N(A)σ≤A+τv−b,            σl≤σ≤σu,

where **f** is the (*n* − *m* × 1) linear objective function vector, **b** is the (*n* × 1) vector that holds minimum allowed positive tensions (lower boundary for **t**), and **σ**_*l*_ and **σ**_*u*_ are, respectively the lower and upper boundaries for the arbitrary **σ** vector in (16). This optimization procedure, when converges to a minimum solution, produces point-wise feasible positive cable tensions that are equal or higher than the limits specified in **b**. However, these feasible cable tensions are not guaranteed to be continuous in time during the task. This discontinuity is not desirable since it may cause instability during real-time control of the robot. Therefore, we introduce a new optimization scheme that will result in cable tensions that are both point-wise feasible and continuous in time. The proposed optimization scheme makes use of the previous solution to be able to choose the current solution to be as close to it as possible. This scheme which minimizes the norm of the difference between the previous and current solution can be formulated as follows:

(18) min⁡σi ⁡||ti−ti−1||,such  that ti=Ai+τvi+N(Ai)σi,   ti−1=Ai−1+τvi−1+N(Ai−1)σi−1,             t0=b   −N(Ai)σ≤Ai+τvi−b,        σl≤σi≤σu.

In order to minimize this constrained nonlinear multivariable objective function, a numerical method can be applied. In this study, the built-in MATLAB (The MathWorks, Inc.) function *fmincon*() is used for that purpose. The effect of using the previous solution on the acquisition of positive cable tensions with the above formulation is discussed after the control problem is addressed.

### 5.3. Trajectory Control with Feedback Linearization

In this section, we solve the control problem for the RLS by using feedback linearization technique. Feedback linearization control, also known as computed-torque control, aims to cancel the nonlinearities of a system and reduce it to a linear system to be controlled by a linear servo law. Decomposing the controller design into model-based and servo-based portions helps solve the control problem in a more systematic way. Model-based portion contains a model of the nonlinearity and includes system parameters. Servo-based portion includes only the control law and is independent of the model-based portion and, therefore, system parameters [15]. The dynamics equation for the RLS is

(19) M(q)q¨+V(q,q˙)+G(q)=τv,

where **τ**_*v*_ = **A** **t** is the virtual input to the system which was previously introduced as an intermediate variable. This virtual input is utilized to be able to find positive and nonzero cable tensions. The model-based portion of the controller is defined as

(20) τv=ατv′+β,

where

(21) α=M(q),β=V(q,q˙)+G(q).

The servo-based portion that includes a proportional-derivative control law is

(22) τv′=q¨d+Kpe+Kde˙,

where ${\ddot{q}}_{d}$ is the desired accelerations, **K**_**p**_ and **K**_**d**_ are, respectively, proportional and derivative control gain matrices. The control gains are both 15 × 15 and diagonal matrices which implies that the PD control law is implemented independently for each degree of freedom (i.e., angle of rotation). **e** = **q**_**d**_ − **q** is the servo error between desired and actual trajectories. The error dynamics of the proposed control law can be found by first plugging (22) into (20) and the resulting equation into the dynamics equation:

(23) q¨=q¨d+Kpe+Kde˙.

Noting that $\ddot{e}={\ddot{q}}_{d}-\ddot{q}$ above equation written in error space becomes

(24) e¨+Kde˙  +  Kpe=0.

The equation above is a second-order differential equation and the coefficients now can be chosen to shape the dynamic response of the system. It should also be noted that the left-hand side of the equation must be a Hurwitz polynomial to provide a stable closed-loop response.

**Figure 4:**
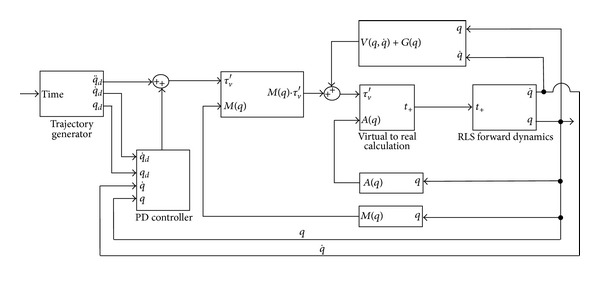
RLS controller schematic.

Controller architecture for the RLS is shown in Figure 4. It is composed of a trajectory generator, PD controller, virtual to real calculation, and the forward dynamics blocks. Trajectory generator provides the desired angles (**q**_**d**_) at every time step based on a quintic polynomial in order to control first and second derivatives (${\dot{q}}_{d}$, ${\ddot{q}}_{d}$) of the desired angles at the beginning and end of a path segment or trajectory. These derivatives are generally set to be zero to be able to obtain smooth movement of the robot. PD controller, as discussed previously, is needed to control the robot to follow the trajectory with a diminishing error between actual and desired angles of rotation. Controller gains can be chosen to obtain desired dynamic response, and they affect the error dynamics, that is, how fast the robot can recover from an error at any given time during the task. Virtual to real calculation is necessary in order to acquire positive nonzero cable tensions (**t**_+_). The inner workings of this block were detailed previously in the section that describes the cable tension optimization.

### 5.4. Simulation Results

The simulations were run for six different motion types (flexion/extension, right/left lateral bendings and right/left torsions). Due to space considerations, however, the results for only (right) lateral bending motion are presented. The results for remaining motion types can be found in [10]. Lateral bending is one of the most involved motion types in terms of the existence of coupled movements. Coupled movement of vertebrae occurs when the motion to the lumbar spine is induced in one specific plane (in frontal plane for lateral bending) which causes vertebrae to move in more than one plane. The desired angles of rotations (Table 1) for the RLS are acquired from experimental data [5] which were obtained after applying 2.5 Nm pure moment about *z*-axis of the freshly frozen lumbosacral spine specimens. The masses of the vertebrae from L5 to L1 are 0.0125, 0.0132, 0.0123, 0.0113 and 0.0100 kg, respectively. The trajectory generator starts at 0.1 sec, and simulation is run for a total of 1.5 sec. The control gains used are 5 and 6 for **K**_**p**_ and **K**_**d**_, respectively.

**Figure 5:**
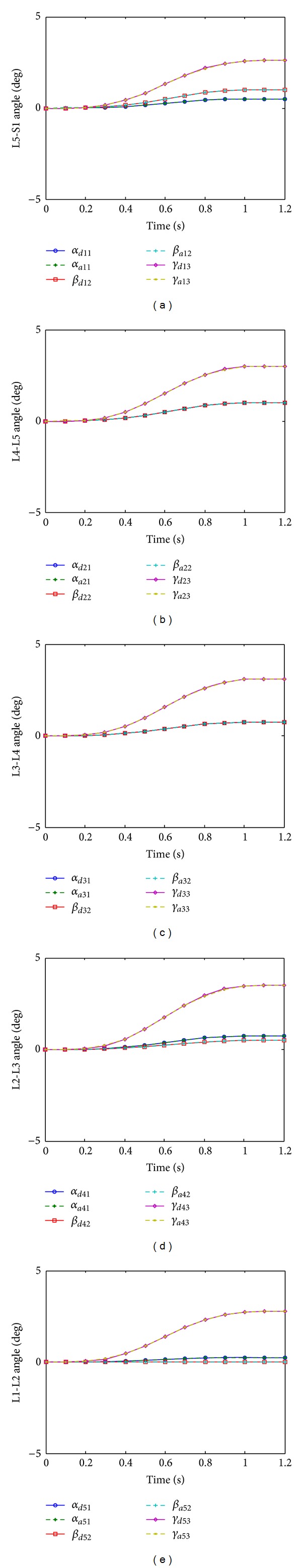
Desired and actual angles of rotations (right bending, *α*_*d*_, *β*_*d*_, *γ*_*d*_: desired, *α*_*a*_, *β*_*a*_, *γ*_*a*_: actual angles of rotation about *x*-, *y*- and *z*-axes, resp.).

**Figure 6:**
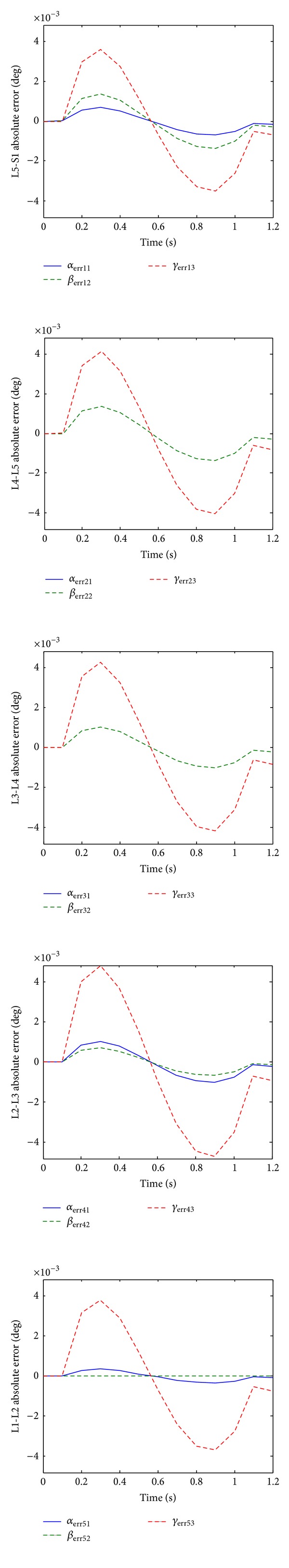
Tracking errors (right bending, *α*_*err*⁡_ = *α*_*d*_ − *α*_*a*_, *β*_*err*⁡_ = *β*_*d*_ − *β*_*a*_, and *γ*_*err*⁡_ = *γ*_*d*_ − *γ*_*a*_).

Figure 5 shows the commanded (desired) and actual paths followed by the RLS. The corresponding tracking errors are shown in Figure 6.

**Figure 7:**
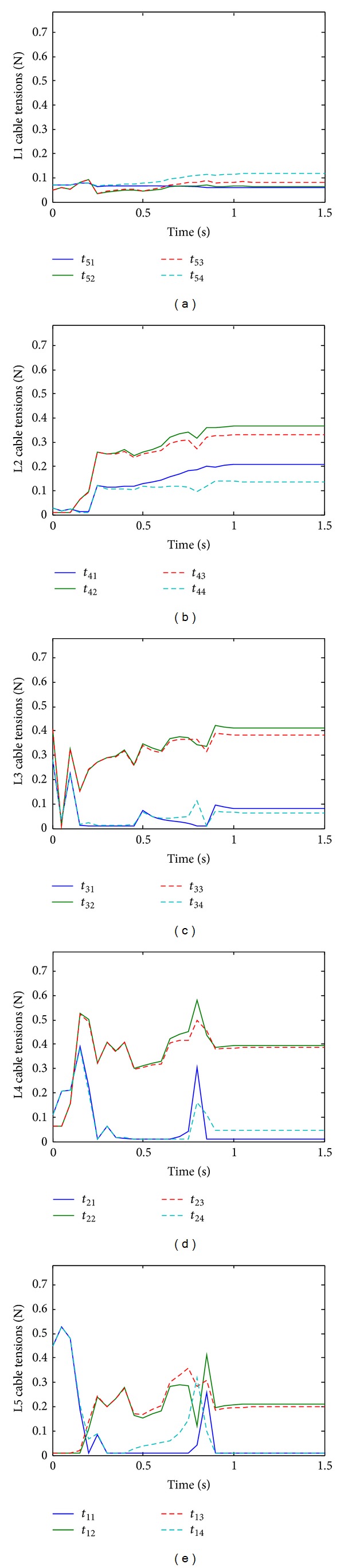
Cable tensions solved without continuity algorithm (right bending).

**Figure 8:**
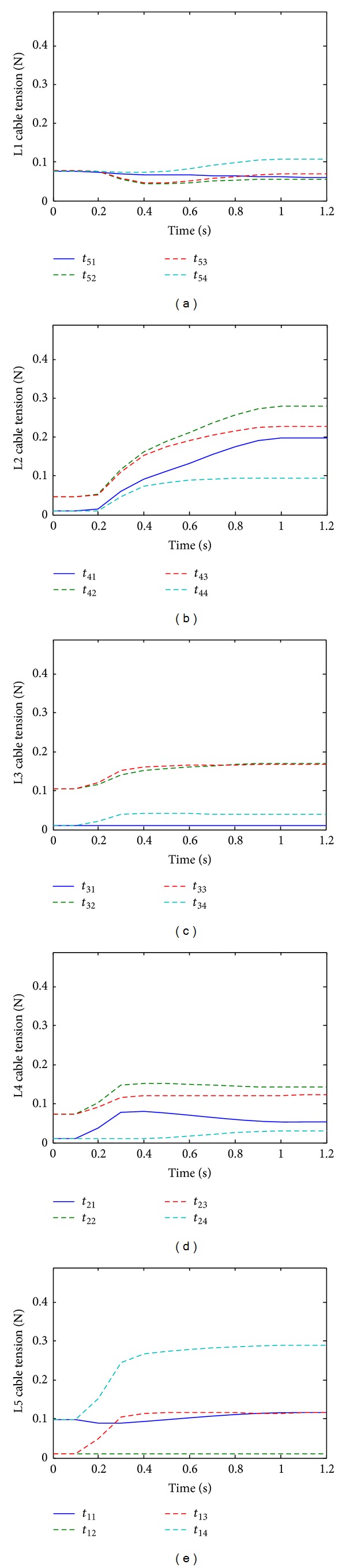
Cable tensions solved with continuity algorithm (right bending).

In order to test the effectiveness of the new method for solving positive continuous cable tensions, the cable tensions are solved with and without implementing the proposed continuity algorithm.  Figure 7 shows the cable tensions without the continuity algorithm. Even though all tensions are solved to be positive discontinuity is apparent.  Figure 8 shows the results of the simulation with the same parameters before but with the continuity algorithm. It is seen that the solved tensions are all positive and continuous in time.

## 6. Discussion

Conceptually, the RLS was designed to support some apparent needs that the instructors and the students of institutions that teach palpatory diagnosis are currently facing. These needs can be collected under three main items:

1. limited variation of dysfunctions that can be practiced in a lab environment,
2. repeatability issues due to the inherent characteristics of tissues to change properties due to repetitive manual manipulation,
3. lack of an objective assessment tool to evaluate the transfer of practical knowledge from the instructor to the students.

With the RLS, there would be virtually no limit to the abnormal movement patterns to practice. These abnormal movement patterns could be programmed easily if the data are readily available, that is, if experimental data or accurate models exist. If no data are available, experience of professional experts may be utilized to generate the required data for abnormal movement patterns by trial and error until a general consensus among the experts is reached. The RLS, as any other robot, would be repeatable (to a certain degree that needs to be calculated and validated experimentally) by configuring itself correctly according to the user's input from the joystick/haptic device. As mentioned previously, there exists no assessment device for instructors to objectively evaluate progress and success of the students in real-life situations. By means of the RLS, all students can be objectively tested on identifying the normal/abnormal movement patterns of the lumbar spine. Since the RLS is also repeatable, any number of students may be tested for the same or different dysfunctions as needed. 

The equations of motion were complex and highly nonlinear. This is expected due to the number of degrees of freedom considered (15 DOFs) and the actuation redundancy. Note that, for systems the links of which are actuated at the joints, **τ** in (1) is independent of the generalized coordinates (*q*) when they are defined to be the joint variables (angle of rotation for revolute and distance for prismatic joints). However, as shown in the text, **τ** for the robotic lumbar spine is a function of the generalized coordinates since the actuation is not performed at the spherical joints. This adds to the complexity of the equations.

The simulations for the control of the RLS showed that the tracking errors were less than 0.005 degrees for all degrees of freedom during the entire range of motion which implied that the designed controller performed as expected. The results of the simulations also showed that the new method proposed to solve for positive cable tensions was very effective in eliminating the spikes in the cable tensions. The results for all motion types, when the effect of the continuity algorithm was tested, were very similar to the results of the right lateral bending as presented here.

The RLS was designed to change configuration by a force-feedback joystick or an affordable haptic device (such as Falcon from Novint Technologies Inc.). By moving the joystick, the angles of rotations will be commanded to the RLS, therefore representing a normal lumbar spine movement. A static model of the human lumbar spine was derived to obtain these normal movement patterns of the lumbar spine for six different types of motion. It is also planned that some abnormalities consistent with known dysfunctional movement patterns (vertebral fusion, rotational resistance of vertebra about an axis, etc.) could be mimicked based on these normal movement patterns.

## 7. Conclusion

The dynamic model and nonlinear control of a 15-degree-of-freedom, cable-actuated robotic lumbar spine (RLS) were presented. A new method was proposed that enables the solution of positive and continuous cable tensions for cable-actuated robots. The simulation results confirmed that the tracking errors during the simulated motion were small and the proposed continuity algorithm proved to be very effective in obtaining positive cable tensions that are also continuous in time.

## Figures and Tables

**Table 1:** Desired angles of rotation for right bending [5].

Motion segment	*α* (°)^†^	*β* (°)^†^	*γ* (°)^†^
L5-S1	0.50	1.00	2.60
L4-L5	1.00	1.00	3.00
L3-L4	0.75	0.75	3.10
L2-L3	0.75	0.50	3.50
L1-L2	0.25	0.00	2.75

^†^ *α*, *β* and *γ* are the angles of rotations about *x*-, *y*-, and *z*-axes, respectively.
